# Assessing Individual Intellectual Output in Scientific Research: Mexico’s National System for Evaluating Scholars Performance in the Humanities and the Behavioral Sciences

**DOI:** 10.1371/journal.pone.0155732

**Published:** 2016-05-19

**Authors:** Eugenio Frixione, Lourdes Ruiz-Zamarripa, Gerardo Hernández

**Affiliations:** 1 Department of Cell Biology, Center for Research and Advanced Studies IPN (Cinvestav), Mexico City, Mexico; 2 Section of Methodology and Theory of Science, Center for Research and Advanced Studies IPN (Cinvestav), Mexico City, Mexico; Katholieke Universiteit Leuven, BELGIUM

## Abstract

Assessing the research of individual scholars is currently a matter of serious concern and worldwide debate. In order to gauge the long-term efficacy and efficiency of this practice, we carried out a limited survey of the operation and outcome of Mexico’s 30-year old *National System of Investigators* or SNI, the country’s main instrument for stimulating competitive research in science and technology. A statistical random sample of researchers listed in the area of Humanities and Behavioral Sciences—one of SNI’s first and better consolidated academic divisions comprising a wide range of research disciplines, from philosophy to pedagogy to archaeology to experimental brain research—was screened comparing individual ranks or "Levels of distinction" to actual compliance with the SNI’s own evaluation criteria, as reflected in major public databases of scholarly production. The same analysis was applied to members of a recent Review Committee, integrated by top-level researchers belonging to that general area of knowledge, who have been in charge of assessing and ranking their colleagues. Our results for both sets of scholars show wide disparity of individual productivity within the same SNI Level, according to all key indicators officially required (books issued by prestigious publishers, research articles appeared in indexed journals, and formation of new scientists), as well as in impact estimated by numbers of citations. Statistical calculation from the data indicates that 36% of members in the Review Committee and 53% of researchers in the random sample do not satisfy the official criteria requested for their appointed SNI Levels. The findings are discussed in terms of possible methodological errors in our study, of relevance for the SNI at large in relation to independent appraisals, of the cost-benefit balance of the organization as a research policy tool, and of possible alternatives for its thorough restructuring. As it currently stands SNI is not a model for efficient and effectual national systems of research assessment.

## Introduction

In today's knowledge-based societies within an intensely competitive global economy, and in the face of increasing environmental and cultural challenges, scientific and technological research has become a topic of national security for most countries, both large and small. As a consequence the evaluation of research, previously a subject of interest restricted to scholarly circles, has turned into a pressing priority for governments, universities, scientific institutes, and industry leaders. Upon its practice now depend major decisions on national policy and enterprise management, preferential funding for academic departments and disciplines, faculty recruitment and grant awarding, and of course the researchers' own personal choices for career advancement. Not surprisingly, therefore, research assessment—always a controversial subject—has itself become a progressively active field of research in recent years, with a vibrant debate about the reliability and effects of the different methods used to estimate the impact and relevance of scientific investigation.

An area of particular concern at present time is the evaluation of individual performance, for it affects not only the researchers’ attitude regarding their personal work but also, collectively, the ranking and therefore the funding opportunities of the organizations to which they belong. The dispute is largely centered on the relative advantages and disadvantages of two main approaches to assess research production [1–4]. One is the traditional peer-review system in which a panel of seasoned experts in a discipline examines the curricula of their colleagues and decides on the value of their achievements. This classic procedure, sometimes dubbed the “Mandarin system” [5], provides in principle the best possible assessment of scholarly productivity but is also liable to subjectivism in judgment, including nepotism and other forms of discrimination either in favor or against examinees, problems that in fact have been found by a number of studies [6–8].

On the other hand bibliometrics, now widely and quickly available as a result of computer-assisted algorithms along with the multiplication of readily accessible databases, is able to measure not only the numbers of papers and rate of publication of any given individual but in addition the actual impact of that research in terms of citations. This approach, often referred to also as “webometrics” [9], complemented by the more recently developed concept of alternative metrics or “altmetrics”—i.e., analyzing the impact of scientific papers on social networks of colleagues [10–13]—, offers quantitative data with the obvious advantage of time and cost savings. The downside is that such mechanical screening often fails to distinguish personal from group or in-drove contributions, neither does it differentiate between disciplines or fields within a discipline that in many cases require dissimilar amounts of labor, or have contrasting speeds of production or patterns of publication. In particular, automatically calculated numbers like the so-called “impact factor” have been heavily criticized for distorting the very essence of scientific work [14–20]. Thus the whole issue of bibliometrics still requires an in-depth technical analysis for fair and proper usage [21].

Criticism pointing to adverse repercussions of excessive research evaluation with either system is indeed justified. Scientists often get discouraged from tackling important though difficult or risky innovative projects in which first-rate publications may take long to come out, if they do so at all. Instead, authors may prefer to work on populous areas of research in which relatively fast results and sizable numbers of citations are to be reasonably expected, rather than pioneering new fields with uncertain outcomes and few other insiders who might refer to their findings. Furthermore, with an eye to journal ranking in the publishing market, editors may feel biased in favor of manuscripts on fashionable topics and against those on untrendy ones, irrespective of their intrinsic scientific value.

Moreover, such scoring procedures also are swaying the scholarly community worldwide in a number of strange ways. In the quest of collecting more citations to their work, researchers are developing strategies of submitting a given manuscript to several journals in tandem as a means of improving its quality along the way [22], and articles have appeared about factors that enhance visibility, impact, and success in the academic job market [23–25], or on how the selection of research products may maximize the ratings of universities [26].

More troubling deviations from a healthy academic behavior are taking place in some emerging economies. Thus, for example, faced with climbing pressure to publish in high-impact international journals, scientists in China universities may grossly exaggerate their findings, or are even willing to pay substantial fees in order to be included as co-authors of papers for which they never contributed anything, thus breeding a profitable business of brokerage in false academic authorship [27].

Consequently, the debate about the reliability and effects of the different methods used to estimate the impact and relevance of research has escalated into new heights of contention [28–36]. Hence, concerned groups of scientists have issued calls for prudence, like the *San Francisco Declaration on Research Assessment* (*DORA*) [17] and the *Leiden Manifesto for Research Metrics* [37] (see also [19, 21]), urging colleagues and institutions to avoid the use of purely quantitative factors for rating the quality of individual papers or their authors, and such statements are being endorsed by scientific organizations, editors and publishers of scholarly media in diverse disciplines.

Additional issues arise when it comes to assessing individual scholars in multiple institutions nationwide, especially if they work on diverse areas of knowledge, as practiced in some countries (reviewed in [38, 39]). A long-lived and remarkable example of this type of mechanism is Mexico’s multifarious *National System of Investigators* (*Sistema Nacional de Investigadores*, hereafter SNI; see Background below). Created by the government in 1984 and thus over 30 years old already, SNI is presently a part of the National Council for Science and Technology (Consejo Nacional de Ciencia y Tecnología, hereafter Conacyt), being officially designed to “recognize, as a result of evaluation, the quality of scientific and technological research…as well as innovation produced in the country, and in this way contribute to promoting and strengthening the quality of research and the training and consolidation of researchers with scientific and technological knowledge at the highest level” [40] (article 3); [41] ("Objetivo").

Positions on the SNI value and on its cost-benefit ratio are as wide-ranging as the research specialties it hosts (for a recent three-way exchange of views on the issue in English see [42] debating another paper [43], which also contains the reply by the latter authors plus a commentary from a third party). Hence opinion polls and formal multilateral meetings have been repeatedly organized across Mexico’s academic community by both, SNI authorities and independent groups of researchers, in the hope of reaching a more agreeable formula for the operation of this complex machinery. Despite frequent official talk that a thoroughly revised framework for national research assessment is already in the offing, however, years and government terms have passed by still using essentially the same original design with only minor adjustments. Thus the relevance of SNI and its actual role as an instrument of national policy remain dubious. Surprisingly, over these many years of existence and dispute only a handful of diagnostic quantitative studies have been published on the SNI [44–49], and none of these has inspected the actual correlation of SNI membership to compliance with official requirements by members.

One reason behind this lack of information about such a critical matter is related, of course, to the difficulties, amount of labor and time associated to examining the production of statistically significant numbers of scholars in a vast assortment of specialties across many scientific disciplines. Nevertheless, a workable starting glimpse of the overall situation can be attempted by restricting the inspection to just one of the SNI’s general areas of knowledge that by official design is itself widely diverse in disciplines and specialties, resembling in this regard the SNI’s full universe. Such endeavor provides an opportunity to gauge the long-term efficacy and efficiency of regularly assessing the research of individual scholars at a national scale.

With this admittedly limited and approximate approach, here we report a quantitative analysis of the correspondence between individual SNI ranks and scholarly production, in terms of the SNI’s own evaluation criteria, for a random sample of professors and a Review Committee belonging in Area IV, which incorporates the humanities and the behavioral sciences. Because it congregates a mixed gamut of academics from philosophers to archaeologists to psychologists doing experimental brain research, Area IV is arguably the broadest in coverage and most heterogeneous of all seven, and in this sense the more analogous to the SNI as a whole.

Data were retrieved from the SNI’s own files, especially released for the purpose of this study, as well as from major databases of scholarly publications. Because our survey comprises careers in all stages of development at multiple research institutions throughout the country, the findings provide a snapshot of SNI’s actual results after 30 years of operation in one of the original and better consolidated divisions of the whole organization. The results are discussed in terms of their reliability, accordance to independent official appraisals, and relevance for the country’s pressing needs in scientific and technological development, within the context of present worldwide rethinking of research assessment. A preliminary pilot test of this project has been locally published [50].

## Background

In awareness of the diversity of scientific disciplines and styles of research, SNI is internally divided into seven general areas of knowledge (Table 1)—upward from the original four (currently areas I, II, IV, and VII)—, each with its own Review Committee constituted by 14 notable academics specialized in particular fields of research within the corresponding general area of knowledge. All seven Review Committees are partly renovated on a yearly basis, so as to always include a mix of new members with veterans in the group.

**Table 1:** SNI Areas of Knowledge.

Area	Name	Current Fraction %
I	Physics, Mathematics and Earth Sciences	15
II	Biology and Chemistry	17
III	Medicine and Health Sciences	10
IV	Humanities and Behavioral Sciences	15
V	Social Sciences	15
VI	Biotechnology, Agriculture and Livestock Sciences	12
VII	Engineering	15

The Review Committees are in charge of devising and revising specific criteria for evaluating the performance of their colleagues in the respective areas of knowledge, according to their own distinct working methods and traditions. Based upon these specific criteria, the SNI Review Committees discuss and assess the individual applications received for each period of evaluation. Applicants are thus appointed as National Investigators in one of four academic ranks or “Levels of distinction”: Candidate (beginner), I (junior), II (intermediate), and III (senior, Emeritus, or occasionally young outstanding) scientist. Through this process over 21,000 scholars currently enrolled in SNI—about half of all now active in Mexico—are periodically evaluated, with mandatory reviewing intervals established according to their SNI Level and the date of their most recent appointment.

Admission into each of these Levels is automatically accompanied by a commensurate and tax-free monthly stipend that may represent up to 30% of the total income for researchers appointed to the upper SNI Levels. Belonging to SNI, as well as the rung attained in its ladder, may be crucial also for scholars getting promoted in their own departments, and for their research to be funded by Conacyt and other supporting agencies. Understandably, few full-time researchers can afford or voluntarily opt for staying independent of SNI, although many are kept out simply because they do not satisfy the quality or productivity requisites.

Claimants who complain of unfair judgment are entitled to appeal, immediately after the results of the contested evaluation become known, for consideration by a separate Revisory Committee in the same area of knowledge. The outcome of applying for this right, however, is final though it seldom meets the plaintiffs’ expectations. Therefore, charges presented by unsatisfied or rejected applicants, either as press releases or in the form of some legal action before various government instances, are also numerous and take up considerable time of Conacyt’s law department.

## Methodology

### Sampling

The extent of compliance with the required official criteria of scientific and academic performance was inspected in two separate sets of researchers who are currently members of SNI Area IV: a) the 14 members of a recent (2012) Review Committee of this Area, and b) a random sample of 58 academics picked from the 2508 members of SNI registered in Area IV that year. Sample size for this second set was defined upon consideration of established theoretical standards [51], of previous results with a pilot test [50], and of a reasonable compromise between reliability of statistical significance and viability for a detailed examination of various kinds of scholarly products from the total 72 researchers surveyed at the individual level.

All sampled researchers were identified in the databases by their family names, a task much easier and safer with Latin than with Saxon or Asian surnames because authors from countries speaking Spanish or other Latin languages commonly sign their publications with both the paternal and maternal family names, usually linking them with a hyphen to further avoid the ambiguity of identity that has become a problem in scientific publishing until quite recently [52]. Looking up the particular fields of work from book or article titles immediately dispelled possible doubts, if any.

Scientists of different ages and therefore diverse academic life spans are included in each of the above two sets, yet the career-length factor was disregarded for the present analysis just as it is also largely done in the SNI regular evaluating exercise. Life production is officially taken into account only when first entering into SNI, or when re-entering after an interruption in membership for any reason. All sampled scholars have been evaluated by Review Committees according to criteria that have changed little with time, since they constitute the classic outcome expected from researchers virtually everywhere (see Academic Products Inspected below). Candidates were not included in our study because this SNI Level is officially stipulated as transitory, with allowance for only one year extension after the first appointment, so it cannot be taken as a relatively stable bracket like the main three Levels.

The first of these two sets of researchers is expected to be representative of academic excellence, given the careful multi-step procedure followed for designating members of the SNI Review Committees. Eligibility to participate in any of such elite groupings requires holding an appointment as National Investigator either Level III or Emeritus, i.e., the top echelons in the hierarchy. Moreover, the statutes of the system demand that the composition of each Review Committee should aim at attaining the best possible “equilibrium and parity among disciplines, institutions, genders and regions” [40] (article 13). Hence, in broad terms a Review Committee may be taken as a model of Mexico’s ideal body of researchers in the main disciplines pertaining to their corresponding general area of knowledge. Table 2 shows the disciplines officially comprised in SNI Area IV: the Humanities and Behavioral Sciences.

**Table 2:** Disciplines in SNI Area IV.

Anthropology	Linguistics
Archaeology	Mathematical Education
Architecture	Pedagogy
Arts and Literature	Philosophy
History	Psychology
Library Sciences	Science Teaching

Although the members of a Review Committee are representative of the spectrum of disciplines normally encompassed in the respective SNI Area, not every one of these disciplines is necessarily represented in a given Review Committee of that Area. In these cases, applications coming from the absent disciplines are turned over to specialists selected by the Committee in request of expert opinions, which the Committee then considers in order to reach decisions. The disciplines represented by the members of the particular Review Committee here inspected are as follows: Anthropology (3), Architecture (1), Arts and Literature (3), History (2), Linguistics (1), Mathematical Education (1), Pedagogy (1), Philosophy (1), Psychology (1).

Our second set or random sample, in turn, is representative of the academic sector that cultivates the disciplines included in SNI Area IV nationwide. Members of this Area may be active in any of the 12 different disciplines, but seven of these account for 96.7% of the 2508 individuals actually registered in this Area, according to the SNI database for 2012. Those disciplines are represented in our random sample in the following percentages, in relation to their distribution at large (numbers in parentheses): Anthropology, 15.5 (16.9); Arts and Literature 13.8 (16.7); History 27.6 (26.7); Linguistics 5.2 (5.5); Pedagogy 5.2 (10.5); Philosophy 12.1 (7.7); and Psychology 12.1 (12.7). The fractions of the total random sample by SNI Levels are as follows, also in reference to their overall proportions (in parentheses): Level I, 63.8 (65.4); Level II, 24.1 (24.5); and Level III, 12.1 (10.1). Further, these researchers show comparable gender ratios (41 women/59 men in the sample vs. 49 women/51 men overall), and are distributed in 23 research institutions throughout 15 of the total 32 federal entities.

### Academic Products Inspected

Our survey focuses on the major *Internal Evaluation Criteria* [53] (hereafter *IEC*; Table 3) applied by the Review Committee of Area IV for deciding admittance or re-admittance of applicants, as well as for appointing their respective SNI Levels. The relevant “products” considered in such criteria fall under two main categories: 1) research results shown as formal documents, i.e., books, articles, book chapters, patents, innovations, technological developments and/or transfers; and 2) proofs of formation of new scientists, i.e., junior researchers trained, theses supervised to students, courses dictated at university level, and research groups formed.

**Table 3:** Internal Evaluation Criteria (*IEC*) in SNI Area IV.

Products	Main Elements Evaluated	Evidence	Ref. items^1^
	specialized scholarly books^2^	copies	III, 3.1.a; 3.2.a
New knowledge (research)	articles in specialized journals^3^	copies / reprints	III, 3.1.b; 3.2.b
	chapters of multi-authored books	copies / reprints	II, 7; 3.1.c
	citations	lists of references	II, 11
New scientists (training)	theses supervised to students^4^	official front pages	III, 3.3.a, b
	teaching at university level	course descriptions	III, 3.3.c
	graduates members of SNI	list of names	III, 3.4.b

^1^ Referred by Section, Number, and Item in the progressive listing of the current official *IEC* document.

^2^ Formally distributed by publishers with widely recognized academic prestige.

^3^ Especially journals indexed in the *Journal of Citation Reports* (*JCR*).

^4^ Especially theses that have led to graduation of PhD candidates.

The paragraphs below particularize the specific sources of information from which data were retrieved, and how the products were appraised according to the respective specific evaluation criteria for SNI Area IV.

### Overall Individual Scholarly Production

The research and new-scientists production of all SNI members here inspected was individually scanned, working out from their own statements as personally registered in the SNI’s *unified-format curriculum vitae* (CVU) database of the whole population of researchers appointed to Area IV, which was obtained directly from SNI by special request for the purpose of this analysis. SNI insists that every member should keep her/his CVU permanently updated, and this requirement becomes imperative in particular for all applicants every year. Thus the CVUs used here should be as accurate as they ever get, and in principle not too different from those that were available to the Review Committees involved in the most recent assessments of these scholars. Still, marked contrasts between data registered in the CVUs and those found in publicly available databases are common rather than exceptions, so a special effort was carried out to locate all products that do comply with the requisites defined in the *IEC* of SNI Area IV as collected from the various databases, occasionally resulting in numbers exceeding those stated by the researchers themselves in their CVUs.

The numbers listed in the tables are actual raw data for each individual scholar here inspected, so as to make possible additional independent calculations or analyses in every case apart from those presented in this paper. Most of such data may vary with time, of course, as a result of the dynamic nature of the databases. Some of these are not only permanently updated but they also progressively incorporate data from past journal editions, so that a relatively old article presently absent in the listings may appear included later on. Similarly items "in press", which legitimately count as finished products, will not appear listed in the databases. Hence, the values here reported are reliably indicative for concurrent internal comparison rather than absolute. Numbers in boldface characters in the tables identify the best overall performers in each group of researchers for every kind of academic product. Patents, innovations, and technological developments were not inspected because their numbers are negligible in the disciplines included in SNI Area IV.

### Research Published in Specialized Scholarly Books

Authorship of specialized research books, including critical editions and annotated translations of classical texts either in the western languages or from other ancient traditions, issued by prestigious academic publishers with wide distribution and having a proper International Standard Book Number (hereafter ISBN), appears first among the *IEC* of SNI Area IV (item III, 3.1.a). Editing or coordinating collective volumes, as well as authoring textbooks or publishing modern critical editions and book translations, are officially taken into account only as “complementary” products for assessing applicants performance (*IEC* items III, 3.1.e; IV, 4.1. Level I.b and Level II.a).

Despite this repeated clear distinction in intrinsic value of the two classes of academic work, however, many CVUs include the latter complementary products in the specialized research book category slot, along with book chapters, prologues and introductions, texts published in conference proceedings, edited volumes of journals, anthologies, common translations (e.g., from English into Spanish), and even audiovisual works in DVD format, some of which show an ISBN. We therefore decided to disaggregate the items that are clearly products of research, as specifically stated in the *IEC*, from all those also registered as books by the authors themselves. The only exceptions to this procedure were special textbooks designed by pedagogues or psychologists, and DVDs produced by artwork researchers.

Such disaggregation of valid products was carried out by first locating every item listed as a book in the CVUs through well-known powerful search engines of general inventories of books (WorldCat.org, Amazon.com, BookFinder.com), and occasionally by looking up the catalogs of the respective publishers. Retrieved items showing bibliographical information consistent with that registered in the CVUs themselves, and also meeting the requisites described in the *IEC* of SNI Area IV, were considered "Valid" and included in the corresponding column of Table 4. Valid titles were then screened for their listing in public academic databases as follows.

**Table 4:** Books Published with ISBN by a Recognized Publishing House.

	Review Committee					Random Sample Level III					Random Sample Level II					Random Sample Level I				
Res.	CVU	Valid	*WCat*	*SPI*	*SPI Ex*	CVU	Valid	*WCat*	*SPI*	*SPI Ex*	CVU	Valid	*WCat*	*SPI*	*SPI Ex*	CVU	Valid	*WCat*	*SPI*	*SPI Ex*
**1**	25	23	23	3	3	19	16	15	14	1	13	10	10	7	2	4	4	4	0	0
**2**	10	6	6	4	1	15	9	9	4	1	3	0	0	0	0	5	4	4	1	0
**3**	**24**	**19**	**19**	**14**	**12**	6	4	3	3	0	9	7	7	5	2	0	0	0	0	0
**4**	28	12	12	1	1	5	5	4	3	0	1	0	0	0	0	2	0	0	0	0
**5**	47	20	20	13	2	15	9	9	6	1	19	5	5	4	1	10	7	7	0	0
**6**	46	10	10	9	3	**30**	**21**	**21**	**10**	**2**	**28**	**17**	**16**	**7**	**6**	5	4	4	4	2
**7**	12	9	9	9	1	41	2	2	2	1	16	7	7	3	0	4	4	4	1	1
**8**	26	16	15	4	2						19	15	15	7	0	4	1	1	1	0
**9**	16	15	15	1	1						2	1	1	0	0	**8**	**6**	**6**	**6**	**4**
**10**	23	20	20	8	6						10	7	7	0	0	1	0	0	0	0
**11**	35	12	12	6	5						14	10	10	4	1	6	2	2	2	0
**12**	16	7	7	1	1						16	10	10	4	2	1	1	1	1	0
**13**	2	2	2	1	1						0	0	0	0	0	8	6	6	6	3
**14**	27	7	6	0	0						11	10	10	1	0	21	15	15	10	0
**15**																4	1	1	0	0
**16**																28	16	15	13	13
**17**																3	2	2	0	0
**18**																0	0	0	0	0
**19**																2	0	0	0	0
**20**																4	0	0	0	0
**21**																2	0	0	0	0
**22**																6	4	4	0	0
**23**																2	1	1	0	0
**24**																2	2	2	0	0
**25**																6	4	4	1	0
**26**																4	1	1	0	0
**27**																0	0	0	0	0
**28**																2	1	1	0	0
**29**																15	7	7	0	0
**30**																2	2	2	0	0
**31**																0	0	0	0	0
**32**																4	2	2	0	0
**33**																5	3	3	0	0
**34**																15	13	13	0	0
**35**																6	1	1	0	0
**36**																38	23	23	2	2
**37**																2	2	2	0	0
**Total**	**337**	**178**	**176**	**74**	**39**	**131**	**66**	**63**	**42**	**6**	**161**	**99**	**98**	**42**	**14**	**231**	**139**	**138**	**48**	**25**
**Mean**	**24.07**	**12.71**	**12.57**	**5.28**	**2.78**	**18.71**	**9.42**	**9.00**	**6.00**	**0.85**	**11.50**	**7.07**	**7.00**	**3.00**	**1.00**	**6.24**	**3.75**	**3.73**	**1.29**	**0.67**

**Res.**, Researcher; **CVU**, total books registered in the *unified curriculum vitae*; **Valid**, valid books according to the *IEC* of SNI Area IV; ***WCat***, books present in *WorldCat*; ***SPI***, books produced by publishers listed in the *Scholarly Publishers Indicators*; ***SPI Ex***, books produced by publishers listed in *Scholarly Publishers Indicators*, other than the home institution.

According to the evaluation criteria of SNI Area IV, the unspecified “prestige” of publishers is taken as a practical cue for appraising the overall quality of the treatises authored by the applicants (*IEC* items II, 4 and 7; III, 3.1.a and c; III, 3.2.a; IV, 4.1. Candidate; 4.1. Level I.b; 4.1. Level III.b; 4.2. Level I; 4.2.N-III.c). Because a publisher’s “prestige” constitutes a largely subjective judgment, this criterion was settled here by the inclusion of its name in the *Scholarly Publishers Indicators* [54] (hereafter *SPI*), i.e., an established ranking of major editorial houses in many countries and several languages, as perceived by a large body of Spanish scholars in the humanities and the social sciences. For chronological consistency we used the original 2012 version of *SPI* that included already the most prestigious Mexican academic publishers—such as *Fondo de Cultura Económica*, *Siglo XXI Editores*, and the *Universidad Nacional Autónoma de México*—, which is still available in the current expanded version of *SPI* (2014) that also contains data from the *Book Citation Index* (Thomson Reuters) and *Scopus Book Titles*.

*SPI* is also useful to reckon the degree of editorial recognition of an author by looking at the number of her or his books issued by *SPI*-listed publishing houses other than those in the respective home institution (i.e., by independent or external prestigious publishers, hereafter *SPI* Ex). Joint productions issued by the author home institution together with an external publisher were not included in this class.

Finally, an indication of the value or usefulness of scholarly books was obtained from their presence in the *WorldCat* [55] global catalog of library holdings (hereafter *WCat*), which reflects acquisition and registration decisions made by members of the international community of scholars and librarians such as those belonging to SNI Area IV itself.

Book chapters were not included in our search because there are no standardized databases to obtain direct quantitative information about their specific relevance, such as for books, articles, and training of other researchers (see below).

### Research Articles Published in High-quality Journals

Original research articles published in specialized high-quality journals are the second prime product used to assess the scientific work of academics in SNI Area IV (*IEC* items III, 3.1.b and 3.2.b). In accordance with those high standards, the immediate recommendation transmitted by the Review Committee to rejected applicants suggests, in the first place, “increasing publications in arbitrated journals of international quality, indexed in the JCR” (i.e., the *Journal of Citation Reports*, hereafter *JCR* or annual ranking of scholarly publications issued by the Thomson Reuters’ Institute of Scientific Information, or ISI).

As in the case of books, the *IEC* of SNI Area IV clearly enunciate those products "which are not taken into account as research", including abstracts, proceedings and memoirs of meetings, or self-edited works (*IEC* item II, 8). Also as with books, however, CVUs show a high incidence of such secondary products, as well as of many other assorted pieces (contributions to institutional gazettes, book prologues and reviews, printed conferences, commentaries, dissertations, notebooks, logbooks, publications in magazines and newspapers or supplements in them, even CDs and DVDs), registered as formal research articles although the CVU layout offers specific slots for at least some of such items that usually remain vacant. A similar miscellany of alleged academic products has been recently documented also for SNI Area V, which assembles the Social Sciences [56].

Therefore the criterion of a journal being arbitrated and “indexed” (*IEC* item II, 7) is here referred to the *Web of Science* [57] database (hereafter *WoS*), i.e., the source upon which the *JCR* recommended by the Review Committee is built. The *JCR* includes or suppresses academic journals in its listings according to their citation profiles every year, so that journals getting too few citations may be absent from the *JCR* at a given moment even though they are indexed in the *WoS*.

In addition we counted papers in publications listed in Conacyt's *Index of Mexican Journals of Scientific and Technological Research* [58], which are classified within the same general areas of knowledge as those of the SNI, plus a multidisciplinary category. The *Index* includes about 140 titles that are periodically selected and validated or discarded according to their overall scholarly quality by a committee created by Conacyt especially for the purpose. Since the second largest number of titles (32) in this *Index* corresponds to journals belonging in the Humanities and Behavioral Sciences, just after that of the Social Sciences (50 titles), many researchers in Area IV seem to have a preference for communicating their research in such select national publications.

### Citations

The number of citations in specialized scholarly literature is a standard indicator of the relative “impact” that a document reporting research has in the corresponding field of science, as specifically recognized by the SNI Area IV (*IEC* items II, 11; IV, 4.1, Level IIIc; 4.2, Level IIIe; see also [59]). In turn, the lifetime number of citations collectively gathered by all works of a scholar may be taken as a meaningful index of the general impact of her/his oeuvre [60, 61]. Hence, we show the total numbers of citations obtained by all researchers here reviewed, as extracted from the *WoS* and *Scopus* [62] databases respectively. In order to include citations to the more recent publications (i.e., up to 2011), this screening was carried out in 2013.

### Formation of New Scientists

Like in all other areas of SNI, involvement of the scholars in the formation of human resources qualified for scientific research, mainly through teaching and supervising doctoral theses that lead to effective graduation but also by acting as co-directors or adjunct advisors of such students, is the most important criterion other than research itself for evaluating the performance of researchers in SNI Area IV (*IEC* items III, 3.3.a, b). Scholarly leadership is to be demonstrated by having trained independent investigators as well as by heading a research group, particularly if the latter has been created by the professor being evaluated (*IEC* item III, 3.4.b).

Since the declared ultimate goal of recognizing the involvement of scholars in the formation of highly qualified human resources is to increase the national strength in competitive scientists and research teams, here we screened the researchers for the numbers of their former students and further academic descendants who also belong now in SNI, down to the third generation in each lineage.

### SNI Level vs. Required Minimal Productivity

For statistical analyses we used a binomial exact test to compare the proportion of researchers who do not comply with the minimum requirements for their SNI Levels, with the null hypothesis Ho = 0.05, using a 0.05 significance level. The 95% confidence interval for this proportion of researchers was estimated using the bias-corrected and accelerated bootstrap method (BCa) [63] with 2000 replicates, through the R boot package [64, 65]. The power of the test was assessed using the formulas [51] (Chapter 8):

$$\delta =2({\text{sin}}^{-1}\sqrt{0.25}-{\text{sin}}^{-1}\sqrt{0.05}=0.596$$

and

$$\Delta =\frac{e^{2\delta }-1}{e^{2\delta }+1}=0.534.$$

## Results

### Research Published in Specialized Scholarly Books

As expected from the superior merit ascribed to this research product in SNI Area IV, the combined total of entries filed by all 72 researchers in the respective CVU slot amounts to 860, i.e., almost 12 per scholar overall (Table 4). This average becomes reduced to about 6.6 once that only products meeting the *IEC*'s standards for research books are counted (columns headed "Valid" in Table 4; see Methodology). Still, over 98% of these valid titles can be found in *WCat*, reflecting their reasonable distribution, availability and use in diverse domestic and foreign libraries around the world. Moreover, average book production per researcher has an appropriate relationship for the respective SNI Levels.

Nevertheless, the total production of research books is strikingly irregular among the individual scholars constituting each group in our random sample, and even in the Review Committee itself where differences between members vary by up to an order of magnitude. Another intriguing finding is that individual totals of authored books drop quite unevenly when they are filtered by the required “prestige” of the respective publishers, as decided merely by the inclusion of the publisher name in the *SPI* listings regardless of its rating; thus, while one member of the Review Committee has managed to get almost 70% of her/his titles published by *SPI*-listed editorial houses, other seemingly productive members in this same set have just one or none books issued by widely recognized publishers. The same pattern was found for all groups in our random sample. In fact, over 40% of all researchers here inspected have no books published by *SPI*-listed editorial houses.

We further enquired into the relative numbers of these books that were published by *SPI*-listed but external editorial houses (*SPI* Ex), i.e., other than those in the authors’ home institutions. Looked at the individual level, just a few researchers have a substantial part of their book production presented by widely recognized independent publishers.

Slight differences in numbers of products officially registered as research books by the scholars, in relation to those considered valid here, may be due to several reasons. For example, our taking out from the counts some useful titles not involving actual or further research, such as second or third printings, common translations of works important for the discipline and the like, as well as books that could not be identified by any of the search engines or in alternative sources because they may be local or private editions with a limited distribution. Sharp contrasts between data in the "CVU" and the "Valid" columns of Table 4, on the other hand, usually result from misfiling of products by the researchers themselves. This includes, for example, the numerous volumes in collections of complete works by classic authors (Researcher 6 in the Review Committee), subsequent issues of journals or other serial publications (Researcher 7 in Level III), and separately published translations of parts of an original complete treatise (Researcher 14 in the Review Committee).

Our analysis also showed puzzling comparisons between colleagues in the same or closely related disciplines but different SNI Levels. One illustrative example involves member 12 of the Review Committee and researcher 16 in SNI Level I, both focused on improving teaching at intermediate school through books that are well represented in *WCat*, and both have been published by the same local branch of a large transnational publishing corporation. Yet the number of valid titles published by the researcher in Level 1 is over twice that of the Review Committee member, with no great differences in productivity of both scholars in other kinds of quality research products (i.e., articles in indexed journals, see below) so as to explain their being appointed at opposite extremes of the SNI hierarchy.

### Research Articles Published in High-quality Journals

Table 5 summarizes the numbers of products registered as “original research results published in arbitrated or indexed journals” in the CVUs of all investigators examined in this study. Members of the Review Committee head all groups in terms of collective output of research articles, though individual contributions range widely from 30 to 131 entries so that over 63% (587) of those papers are authored by less than half of the Committee members (researchers 4–6 and 12–14, i.e., 6 out of 14). A still more marked unevenness regarding this criterion among individual numbers is found in terms of papers indexed in the *WoS* or published in journals currently present in the *JCR*, with only one Committee member (number 13) accounting for nearly all credit in this regard. The same pattern of just one researcher having published a sizable fraction of papers visible in the *WoS* and included in journals listed in the *JCR* is found for all three SNI Levels in the random sample of Area IV.

**Table 5:** Articles Published in Arbitrated and Indexed Journals.

	Review Committee					Random Sample Level III					Random Sample Level II					Random Sample Level I				
Res.	CVU	*WoS*	*JCR*	*Scopus*	Conacyt	CVU	*WoS*	*JCR*	*Scopus*	Conacyt	*CVU*	*WoS*	*JCR*	Scopus	Conacyt	CVU	*WoS*	*JCR*	*Scopus*	Conacyt
1	44	11	0	1	8	139	0	0	0	5	36	2	0	0	2	62	0	0	0	1
2	45	0	0	0	3	68	0	0	0	4	**48**	**18**	**13**	**21**	**0**	41	0	0	1	5
3	50	5	4	4	3	41	0	0	0	0	13	0	0	0	0	20	1	1	0	0
4	131	0	0	2	3	13	0	0	0	2	24	0	0	0	5	17	0	0	0	1
5	90	0	0	0	17	32	1	1	6	2	41	0	0	0	4	26	0	0	0	4
6	125	0	0	0	2	61	5	0	0	1	81	1	0	0	14	12	0	0	0	4
7	38	0	0	0	7	**51**	**29**	**29**	**29**	**0**	12	0	0	0	2	19	0	0	0	0
8	43	0	0	0	6						100	0	0	0	0	22	0	0	1	4
9	30	0	0	0	0						29	4	0	4	4	44	1	0	0	0
10	48	1	0	0	1						10	0	0	0	0	9	0	0	0	1
11	40	4	2	0	1						14	0	0	0	4	11	0	0	0	0
12	79	1	0	6	6						109	1	1	0	6	8	1	0	0	1
13	**92**	**35**	**26**	**87**	**0**						32	3	3	8	0	11	2	2	0	0
14	70	0	0	0	0						52	1	0	3	9	35	0	0	0	0
15																13	0	0	0	0
16																55	0	0	0	0
17																23	0	0	0	7
18																5	0	0	0	0
19																17	0	0	0	0
20																11	0	0	0	0
21																62	0	0	0	0
22																12	0	0	0	1
23																3	0	0	0	0
24																15	0	0	0	2
25																13	0	0	0	0
26																14	0	0	0	0
27																**10**	**5**	**5**	**0**	**1**
28																25	1	1	1	0
29																11	0	0	0	0
30																12	0	0	3	0
31																8	0	0	0	0
32																15	0	0	0	0
33																28	0	0	0	0
34																32	1	1	0	0
35																18	0	0	0	0
36																9	0	0	0	0
37																9	0	0	0	0
**Total**	**925**	**57**	**32**	**100**	**57**	**405**	**35**	**30**	**35**	**14**	**601**	**30**	**17**	**36**	**50**	**757**	**12**	**10**	**6**	**32**
**Mean**	**66.07**	**4.07**	**2.29**	**7.14**	**4.07**	**57.86**	**5.00**	**4.29**	**5.00**	**2.00**	**42.93**	**2.14**	**1.21**	**2.57**	**3.57**	**20.46**	**0.32**	**0.27**	**0.16**	**0.86**

**Res.,** Researcher; **CVU**, total articles registered in the *unified curriculum vitae*; ***WoS***, articles published in journals listed in the *Web of Science*; ***JCR***, articles published in journals listed in the *Journal of Citation Reports*; ***Scopus***, articles published in journals listed in *Scopus*; **Conacyt**, articles published in journals included in Conacyt's *Index of Mexican Journals of Scientific and Technological Research*.

Remarkably, it is not the researchers declaring the higher numbers of published papers who also get higher numbers in the *WoS* and *JCR* columns; instead, those who fill these requirements all rank among those declaring medium or medium-high totals of published articles. In contrast, the most productive authors of research articles in each group, according to the CVUs, are characteristically poor performers in the *WoS* and *JCR* columns. Interestingly, the best scores in both *WoS* and *JCR* correspond to researchers that are all psychologists, although not every psychologist seems to be similarly fertile in this kind of product.

It is often argued that research in the humanities and social sciences is misrepresented in databases like the *WoS* and *JCR*, which were born and originally fed from journals belonging in the hard sciences and are written mostly in English. Scientific output in the humanities and social sciences, the reasoning goes, appears largely in books (see above) and in journals published in different languages that lay below or off-limits the radar of automated retrieval systems like those managed by ISI for the *WoS* and *JCR*. Yet the data obtained from Conacyt's own *Index of Mexican Journals of Scientific and Technological Research* do not support this claim for the authors reviewed in the present survey. The numbers of papers published in national journals of recognized quality are far from matching the totals reported in the respective CVUs, even for those cases in which a marked preference for domestic publication is evident, e.g., Committee members 1, 5, 8, and 12; most of the researchers in Level III, and those listed as 6 and 14 in Level II.

A more complete range of academic publications in general can be obtained by looking into the *Scopus* [62] and *Google Scholar* databases, in addition to those belonging to the *Web of Science*. *Google Scholar*, we found, may be useful to get an indication of the overall “visibility” of an author but hardly adequate for the present study because it mixes professional publications (in books, academic journals, etc.) with articles, interviews and reports in magazines and newspapers, none of which are valid for the SNI. Dissecting the scholarly publications from contents in the media would be, apart from laborious, prone to introducing subjective factors in the analysis. *Scopus*, on the other hand, even though combining journal articles with book chapters and other sorts of academic publications, did provide a complementary insight for our study (*Scopus*-labeled columns in Table 5). Nevertheless, the pattern of productivity revealed by *Scopus* is once again highly irregular, typically with only one researcher in each group contributing a much larger share of publications visible in this database, while many of the other colleagues in all groups show zero publications.

Since articles usually take much shorter times for being both completed and then published, in relation to books, their appearance provides a comparatively better temporal resolution of the research activity carried out by a scholar. Accordingly, the *IEC* of Area IV define several specific periods for evaluating the scientific productivity of applicants in different situations (*IEC* items II, 1, 2, and 7). Based upon these intervals, in order to appreciate the more recent situation the production of articles by all groups of scientists here examined is further presented in two time windows (Table 6): a) the total or complete lifetime production; b) the recent production (2008–2011) at the time of our sampling.

**Table 6:** Total and Recent Articles Published in Arbitrated and Indexed Journals.

		Total Articles				Recent Articles (2008–2011)			
Group	n	CVU	*WoS*	*JCR*	Conacyt	CVU	*WoS*	*JCR*	Conacyt
Review Committee	14	925 (100%) Mean 66.07	57 (6.16%) Mean 4.07	32 (3.45%) Mean 2.29	57 (6.16%) Mean 4.07	94 (100%) Mean 6.71	29 (30.85%) Mean 2.07	14 (14.89%) Mean 1.0	2 (2.12%) Mean 0.14
Random Sample Level III	7	405 (100%) Mean 57.86	35 (8.64%) Mean 5.0	30 (7.4%) Mean 4.29	14 (3.45%) Mean 2.0	69 (100%) Mean 9.86	14 (20.28%) Mean 2.0	12 (17.39%) Mean 1.71	0 (0%) Mean 0.0
Random Sample Level II	14	601 (100%) Mean 42.93	30 (4.99%) Mean 2.14	17 (2.82%) Mean 1.21	50 (8.31%) Mean 3.57	113 (100%) Mean 8.07	15 (13.27%) Mean 1.07	12 (10.61%) Mean 0.86	3 (2.65%) Mean 0.21
Random Sample Level I	37	757 (100%) Mean 20.46	12 (1.58%) Mean 0.32	10 (1.32%) Mean 0.27	32 (4.22%) Mean 0.86	241 (100%) Mean 6.51	6 (2.48%) Mean 0.16	4 (1.65%) Mean 0.11	9 (3.73%) Mean 0.24

**Group**, set of researchers inspected; **n**, number of scientists in each group; **CVU**, total articles published by each group as registered in the respective *unified curricula vitarum*; ***WoS***, total articles published by each group in journals listed in the *Web of Science*; ***JCR***, total articles published by each group in journals listed in the *Journal of Citation Reports*; **Conacyt**, total articles published by each group in journals included in Conacyt's *Index of Mexican Journals of Scientific and Technological Research*.

In the more recent years all groups of scholars show a significant increase in the fraction of research articles that got published in journals listed in the *WoS* database, as well as in those also included in the *JCR*. Nevertheless, even those groups with the highest average scores—almost 31% papers in *WoS* and 17% in *JCR*—are still far off the goal of having most of the research results published “in arbitrated or indexed journals.” Moreover, it is evident that neither the members of the Review Committee (with a recent average individual production of only 0.5 *WoS*-listed papers per year, and just half that average in *JCR*-indexed journals), nor the researchers in the random sample groups (which have still lower numbers, except Level III), are even close to meeting the required minimum (if no other research products are presented) of publishing 5 indexed articles in the average 4-year period between evaluations (*IEC* item II, 7). In fact, if all members of the Review Committee would contribute equally to their collective average, at the observed rate it would take each of them nearly 10 years to publish those 5 *WoS*-listed papers.

### Citations

The citation criterion also reveals vast differences in individual contributions to the collective numbers, with three or fewer researchers accounting for virtually all citations in each group, whereas the vast majority of them get hardly any or no mentions in their respective fields of work as reported by the *WoS* (Table 7) and *Scopus* (Table 8) databases. Understandably, it is basically the researchers who publish in *WoS*-listed journals that also get cited in this kind of publications. Citation profiles are slightly better for some scholars when they are looked up in *Scopus* in relation to *WoS* (e.g., Committee member 12, researcher 13 in Level II); yet in general our results indicate that Mexican research in this SNI area of knowledge is largely overlooked by the systems tracking impact of published scholarly papers, and accordingly also by the international scientific community.

**Table 7:** Accumulated Citations in Indexed Journals (*WoS*, 2013).

	Review Committee				Random Sample Level III				Random Sample Level II				Random Sample Level I			
Res.	CVU	*WoS*	Times Cited	Cites/ Article	CVU	*WoS*	Times Cited	Cites/ Article	CVU	*WoS*	Times Cited	Cites/ Article	CVU	*WoS*	Times Cited	Cites/ Article
1	44	11	1	0.09	139	0	0	0	36	2	0	0	62	0	0	0
2	45	0	0	0	68	0	0	0	**48**	**18**	**91**	**5.05**	41	0	0	0
3	50	5	2	0.4	41	0	0	0	13	0	0	0	20	1	3	3
4	131	0	0	0	13	0	0	0	24	0	0	0	17	0	0	0
5	90	0	0	0	32	1	1	1	41	0	0	0	26	0	0	0
6	125	0	0	0	61	5	0	0	81	1	0	0	12	0	0	0
7	38	0	0	0	**51**	**29**	**105**	**3.62**	12	0	0	0	19	0	0	0
8	43	0	0	0					100	0	0	0	22	0	0	0
9	30	0	0	0					29	4	6	1.5	44	1	0	0
10	48	1	0	0					10	0	0	0	9	0	0	0
11	40	4	0	0					14	0	0	0	11	0	0	0
12	79	1	1	1					109	1	0	0	8	1	0	0
13	**92**	**35**	**254**	**7.25**					32	3	1	0.33	11	2	0	0
14	70	0	0	0					52	1	0	0	35	0	0	0
15													13	0	0	0
16													55	0	0	0
17													23	0	0	0
18													5	0	0	0
19													17	0	0	0
20													11	0	0	0
21													62	0	0	0
22													12	0	0	0
23													3	0	0	0
24													15	0	0	0
25													13	0	0	0
26													14	0	0	0
27													**10**	**5**	**77**	**15.4**
28													25	1	2	2.0
29													11	0	0	0
30													12	0	0	0
31													8	0	0	0
32													15	0	0	0
33													28	0	0	0
34													32	1	9	9
35													18	0	0	0
36													9	0	0	0
37													9	0	0	0
**Total**	**925**	**57**	**258**	**8.74**	**405**	**35**	**106**	**4.62**	**601**	**30**	**98**	**6.88**	**757**	**12**	**91**	**29.4**
**Mean**	**66.07**	**4.07**	**18.42**	**0.62**	**57.86**	**5.0**	**15.14**	**0.66**	**42.93**	**2.14**	**7.0**	**0.49**	**20.46**	**0.32**	**2.46**	**0.792**

**Res.**, researcher; **CVU**, total articles registered in the *unified curriculum vitae*; ***WoS***, total articles published in journals listed in the *Web of Science*; **Times Cited**, total accumulated citations reported in the *Web of Science*; **Cites/Article**, average citations per article reported in the *Web of Science*.

**Table 8:** Accumulated Citations in Indexed Journals (*Scopus*, 2013).

	Review Committee				Random Sample Level III				Random Sample Level II				Random Sample Level I			
Res.	CVU	*Scopus*	Times cited	Cites/ article	CVU	*Scopus*	Times cited	Cites/ article	CVU	*Scopus*	Times cited	Cites/ article	CVU	*Scopus*	Times cited	Cites/ article
1	44	1	1	1	139	0	0	0	36	0	0	0	62	0	0	0
2	45	0	0	0	68	0	0	0	**48**	**21**	**89**	**4.24**	41	**1**	**2**	**2**
3	50	4	0	0	41	0	0	0	13	0	0	0	20	0	0	0
4	131	2	3	1.5	13	0	0	0	24	0	0	0	17	0	0	0
5	90	0	0	0	32	6	32	5.35	41	0	0	0	26	0	0	0
6	125	0	0	0	61	0	0	0	81	0	0	0	12	0	0	0
7	38	0	0	0	**51**	**29**	**154**	**5.31**	12	0	0	0	19	0	0	0
8	43	0	0	0					100	0	0	0	22	1	0	0
9	30	0	0	0					29	4	12	3	44	0	0	0
10	48	0	0	0					10	0	0	0	9	0	0	0
11	40	0	0	0					14	0	0	0	11	0	0	0
12	79	6	35	5.83					109	0	0	0	8	0	0	0
13	**92**	**87**	**826**	**9.49**					32	8	28	3.5	11	0	0	0
14	70	0	0	0					52	3	2	0.67	35	0	0	0
15													13	0	0	0
16													55	0	0	0
17													23	0	0	0
18													5	0	0	0
19													17	0	0	0
20													11	0	0	0
21													62	0	0	0
22													12	0	0	0
23													3	0	0	0
24													15	0	0	0
25													13	0	0	0
26													14	0	0	0
27													10	0	0	0
28													25	1	3	3
29													11	0	0	0
30													**12**	**3**	**10**	**3.33**
31													8	0	0	0
32													15	0	0	0
33													28	0	0	0
34													32	0	0	0
35													18	0	0	0
36													9	0	0	0
37													9	0	0	0
**Total**	**925**	**100**	**865**	**17.82**	**405**	**35**	**186**	**10.66**	**601**	**36**	**131**	**11.41**	**757**	**6**	**15**	**8.33**
**Mean**	**66.07**	**7.14**	**61.79**	**1.27**	**57.86**	**5.00**	**26.57**	**1.52**	**42.93**	**2.57**	**9.36**	**0.82**	**20.46**	**0.16**	**0.41**	**0.23**

**Res.**, researcher; **CVU**, total articles registered in the *unified curriculum vitae*; ***Scopus***, total articles published in journals listed in *Scopus*; **Times Cited**, total accumulated citations reported in *Scopus*; **Cites/Article**, average citations per article reported in *Scopus*.

Such poor citation levels motivated us to briefly look into the numbers of references that are in turn cited by some of the papers authored by the researchers here inspected. This partial exploration showed that many of those publications appear as citing very few or no references at all, with contrasts among individual researchers being sharp in this regard as well. In summary, our results in this connection indicate that the majority of SNI Area IV members publish articles apparently unrelated to other commonly available research documents elsewhere.

Inspecting a random assortment of such stand-alone publications revealed that they often lack any kind of critical apparatus, other than an occasional reference to the author’s previous work or that of his associates. Many of these documents, registered in the CVUs as articles containing “original research results,” may rather consist of the authors’ own thoughts and views about a topic of their disciplines, prologues for books written by colleagues, texts of seminars or lectures, and even considerations on the relative value of publishing research results in *JCR*-listed journals. There are also quite a few duplications of the same content, sometimes due to re-publication in different languages, though occasionally in the same language but different journals. A related interesting finding in this exercise is that more than one researcher in SNI Area IV (one of them external to the sets here inspected) publishes almost exclusively in a journal of which she/he is the founding and permanent chief editor.

### Formation of New Scientists

Table 9 shows the numbers of post-graduate students supervised in doing research up to PhD graduation, as registered (CVU-labeled columns) by the members of the Review Committee and researchers in the random sample from all SNI Levels of Area IV. Also shown are the numbers of those immediate academic descendants who currently belong to SNI (G1), followed by the numbers of PhDs supervised by the latter that also are today members of SNI (G2), and so on down to the third generation (G3) of SNI members who are academic descendants of the researchers here inspected.

**Table 9:** PhD Graduates and Subsequent Academic Descendants who are Members of SNI.

	Reviewing Committee					Random Sample Level III					Random Sample Level II					Random Sample Level I				
Res.	CVU	PhD	G1	G2	G3	CVU	PhD	G1	G2	G3	CVU	PhD	G1	G2	G3	CVU	PhD	G1	G2	G3
1	49	7	1	0	0	2	2	2	0	0	3	0	0	0	0	29	1	0	0	0
2	15	12	4	0	0	8	2	0	0	0	40	0	0	0	0	**8**	1	0	0	0
3	11	1	0	0	0	34	2	1	0	0	7	4	0	0	0	1	0	0	0	0
4	5	0	0	0	0	13	2	0	0	0	32	1	0	0	0	7	0	0	0	0
5	22	8	4	2	0	19	8	6	1	0	13	5	1	1	0	38	3	0	0	0
6	48	26	10	4	0	46	14	3	0	0	3	2	0	0	0	18	2	1	0	0
7	36	10	5	1	0	69	**12**	**5**	**1**	**0**	25	6	1	0	0	12	3	1	0	0
8	9	1	0	0	0						9	1	0	0	0	11	0	0	0	0
9	26	7	2	1	0						14	1	0	0	0	9	0	0	0	0
10	27	10	1	3	1						7	0	0	0	0	3	0	0	0	0
11	39	2	0	0	0						16	1	0	0	0	49	0	0	0	0
12	8	4	1	0	0						74	**25**	**10**	**5**	**1**	2	0	0	0	0
13	60	**15**	**8**	**11**	**6**						29	5	1	0	0	27	0	0	0	0
14	42	4	2	3	0						18	5	3	0	0	20	7	4	0	0
15																5	0	0	0	0
16																33	9	0	0	0
17																31	2	0	0	0
18																11	0	0	0	0
19																1	0	0	0	0
20																22	0	0	0	0
21																8	0	0	0	0
22																1	0	0	0	0
23																1	0	0	0	0
24																7	0	0	0	0
25																4	0	0	0	0
26																9	0	0	0	0
27																1	0	0	0	0
28																12	3	1	0	0
29																22	3	1	0	0
30																28	4	0	0	0
31																5	0	0	0	0
32																2	0	0	0	0
33																0	0	0	0	0
34																**16**	**4**	**2**	**4**	**0**
35																11	0	0	0	0
36																0	0	0	0	0
37																8	5	0	0	0
**Total**	**397**	**107**	**38**	**25**	**7**	**191**	**42**	**17**	**2**	**0**	**290**	**56**	**16**	**6**	**1**	**472**	**47**	**10**	**4**	**0**
**Mean**	**28.36**	**7.64**	**2.71**	**1.79**	**0.50**	**27.29**	**6.00**	**2.43**	**0.29**	**0**	**20.71**	**4.00**	**1.14**	**0.43**	**0.07**	**12.76**	**1.27**	**0.27**	**0.11**	**0**

**Res.**, researcher; **CVU**, total students supervised up to graduation with any university degree, as registered in the *unified curriculum vitae*; ***PhD***, students supervised up to PhD graduation; **G1**, students supervised up to PhD graduation who currently belong to SNI; **G2**, students supervised up to PhD graduation by the immediate academic descendants (**G1**), and who currently belong to SNI; **G3**, students supervised up to PhD graduation by the second generation of academic descendants (**G2**), and who currently belong to SNI.

It can be seen that all groups of professors have been quite productive in supervising theses to students who finally obtained PhD degrees with such research work. Since academics in higher SNI Levels usually have longer careers, the average numbers of supervised theses grow from just over 1 up to more than 7 depending on the SNI Level. Yet, strikingly, even in the most successful examples less than 40% of those former students are today members of the SNI, and very few of them have in turn trained new SNI members on their own. It is clear from these results, therefore, that guiding students up to graduation as PhDs is not necessarily equal to forming new researchers in this area of knowledge, or to starting lineages of these, as SNI seems to believe.

### SNI Level vs. Required Minimal Productivity

Because the academic productivity found here ranges wide, with a few individuals having produced a considerable amount of work in contrast to other colleagues in the same SNI Level (see Tables above), we finally asked what is the proportion of individuals whose research productivity does not meet the minimum required for the SNI Level assigned to them, according to the *IEC* of SNI Area IV. These specify that an applicant should have produced at least either one book or five research papers in the period evaluated, which is typically of 3 years for most cases except for scholars appointed to Level III who are usually evaluated every 5 years. Since this rule sets both a minimum research production-per-period threshold and an equivalence for these two types of publications, we settled for an averaged convention that a scholar appointed to SNI Level I ought to have at least the research production required for one four-year period; a SNI Level II Investigator must have a total research production corresponding to three of these terms; and a SNI Level III Investigator should have a total research production as expected for four of such periods. According to this reckoning, 36% of the Review Committee members and 53% of professors in the random sample do not satisfy the research amount required for their appointed SNI Levels. Still more intriguing, there are instances (e.g., researchers number 3 in Level III, number 4 in Level II, and several in Level I) that show so few valid products of any type that is difficult to explain how they even became members of SNI.

Although it is difficult to justify that a person could be granted a SNI Level without meeting such minimal requirements, in principle there could be exceptional cases where other considerations might prevail. Therefore, we decided to take as null hypothesis that at most 5% of the researchers failed to produce the demanded productivity. Using the binomial exact test for the 53% of researchers that did not meet such minimum in our sample (for the test, 53% or more), we obtained a *p*-value of 2.2e-16.

The 95% confidence interval for researchers that do not comply with the minimum requirements was estimated as (40%, 65%) by the bias-corrected and accelerated bootstrap method (BCa) with 2000 replicates through the R boot package (see Methodology above).

For the power of the test we took a rather conservative effect size of 25%, that is, we considered as significant that at least 25% of the researchers failed to comply with the productivity requirements, compared to the expected 5% mentioned above. According to the tables given by Kraemer and Blasey [51] (pp. 105–106), to a one-tailed test with 5% level of significance, with Δ = 0.50 and sample size n = 58, corresponds a power of 99%. This means that we obtain a certainty of 99% to determine a true difference with respect to the null hypothesis. Our conservative 25% is well below the 40% established as minimum value of our 95% confidence interval. Therefore we can establish that, considering all our assumptions, the fraction of scholars with research productivity lower that the minimum demanded by the *IEC* of SNI Area IV is above 40%.

## Discussion

The assessment of individual scholars at a national scale, as it is now carried out in some countries (reviewed in [38, 39]), has become a necessity for optimizing the labor of the domestic intellectual workforce according to both ambitious development policies and hard economic realities. Because such a process involves adjusting traditional academic practices, it may take years to become really functional so it is crucial to follow the most efficient course possible in establishing it. In this context the accumulated experience of pioneer systems in this sort of exercise may be helpful. The findings contained in the present report suggest that caution must indeed be taken when designing the structure and methodology of organisms created for periodically evaluating the performance of large numbers of researchers.

As mentioned in the Introduction, Mexico’s *National System of Investigators* (SNI) offers an opportunity to inspect the results of a highly organized assessment of researchers on a regular basis over several decades, with a nationwide coverage across many if not all the scientific disciplines. It must be stressed here, however, that our goal is to examine the long-term outcome of a complex and expensive government agency, according to its own rules and procedures, rather than evaluating the research of the individuals contained in our sampling. Bearing this in mind is important for an adequate outlook of the present analysis.

The most relevant feature in our findings is that, after having been in operation for over 30 years with as many consecutive Review Committees involved, at least one of the SNI's seven general areas of knowledge displays a wholly unintelligible ranking of researchers. Marked unevenness of productivity in terms of the main official evaluation criteria (*IEC*) is observed among individual scholars with equal SNI Levels and therefore supposedly equivalent careers. Such inconsistency occurs even within the Review Committee here inspected, although such select groups are in principle composed of reputedly outstanding and often laureate senior professors.

Unevenness in the yield of specific products is in some cases clearly due to individual preferences for diffusing research work. Thus, for example, the Review Committee member 3 opts mainly for writing books whereas member 13 favors publishing articles, both being obviously prolific authors in their respective disciplines. Other researchers, however, declare high productivities that objective evidence just does not bear out, though the discrepancy may be partly due to misfiling of products in the CVUs as pointed out in the respective subheadings of the Methodology and Results sections. Statistical analysis indicates that up to one third of the Review Committee members and over half of researchers in the other sampled groups may be in fact ineligible for their appointed SNI Levels. Several considerations are in order before extracting useful conclusions from this study.

### Reliability of the Results

A first point to take into account refers to possible sources of error in our methodology, starting from the total of only 72 scholars here examined out from a universe of over 2500 currently registered in Area IV, i.e., about 2.88% of the whole. This is important because of controversies regarding the statistical significance of tests concerning research assessment [66]. Nevertheless, 1% samples are usual and routinely reliable in all kinds of statistical studies [51]. In fact, the size of our random sample (58 scholars) is slightly above the average of 56.57 researchers per general area of knowledge taken for a recent inspection of SNI performance by the government Superior Federal Auditor [67] (hereafter SFA). Also, the magnitudes of dispersion in values within each of all groups here examined make it extremely unlikely that a larger sample would yield different results. Further, given the degree of similarity in general features between our random sample and the complete Area IV (see Sampling in the Methodology section), despite slight deviations in representativeness of at most 5.3% of the whole and just for a couple of disciplines (i.e., Philosophy and Pedagogy), little doubt may remain that the profiles shown here are reliably indicative of this SNI division at large. In fact, a chi-squared test comparing the proportions for the seven most representative disciplines in the sample and the population showed non-statistical difference (p = 0.75, with Monte Carlo simulation, 2000 replicates). This confidence is also supported by the fact that our present results confirm and extend findings of a previous pilot study with professors affiliated to one particular research institute [50].

The use of information contained in the CVUs might be questioned too because, although members are obliged to keep their data updated especially before submitting applications to enter or remain in SNI, there is presently no mechanism to ensure that this requirement is strictly and exactly fulfilled in every case; hence, the CVUs may not be fully reliable for this kind of analysis. We agree, though adding still another issue: according to our observations while inspecting CVUs of researchers in SNI Area IV, the main concern regarding their reliability is related to the often stark disparity of their contents to what can be found in independent sources of information, as already mentioned, rather than to their being outdated or incomplete. For both of these reasons, the data taken from CVUs are included here only as a means to compare how researchers officially present themselves to SNI when registering academic products, in relation to their images as reflected in major databases of scientific publications.

It could also be argued that in actual practice the SNI Review Committees assess researchers on the basis of book, article, and other documents copies submitted along with the applications themselves, rather than by looking up at fluctuating values of perceived publisher prestige and *JCR*-ratings, since this latter procedure would be hardly adequate given the huge numbers of applications processed every year. Yet, apart from the obvious fact that reading copies of the products supplied with the applications would be even more time-consuming than just checking up the ratings, it should be observed that our study also disregards such numerical values. We merely noted whether the book publisher name or journal title was included in the *SPI* or *JCR* listings, regardless of its respective rating, as an objective standard of compliance with the requisites stated in the specific evaluation criteria (*IEC* item III, 3.1.a and b).

As mentioned above, the use of such world-class databases is often charged by scholars in the humanities and the behavioral or social sciences as biased and deceiving, on the grounds that products of their academic disciplines are of interest primarily for Mexican and other Spanish-speaking readerships, rather than for global audiences as is the case with the sciences of nature or engineering. “Regional” instead of “international” interest is the term commonly used by partisans of such criticism when referring to an assembly of states involving more than one country in the limited sense that is claimed; which opens the question of whether findings in one country might not be of interest or perhaps useful for other comparable countries (e.g., emergent or developing) although they may speak different languages.

Anyhow, such contentions are at odds with the contents of the 8-page *IEC* document of SNI Area IV, where the adjective “international” is found 20 times, often in relation to terms like “circulation” and “recognition”, and occasionally associated with “transcendence”, “invitation”, “meetings”, or “prestige”. The argument is inconsistent with the also mentioned official suggestion to rejected applicants of “increasing publications in arbitrated journals of international quality, indexed in the *JCR*”. Moreover, the data obtained from journals included in Conacyt's *Index of Mexican Journals of Scientific and Technological Research* show poor overall performance also in such regional publications. Lastly in this connection and contrary to the position of those critics, value has been found in publicly available databases for adequately gauging the relative merit of research in the social sciences and the humanities [60, 61]. Publishing preferably or solely in Latin American or other regional journals, on the other hand, is prone to high rates of self-citation, restriction to local languages and other endogamous isolating practices, which are detrimental to both a wide projection of the new knowledge and to international scientific communication in general, particularly in the humanities and social sciences (reviewed in [68]).

Finally, the mismatch between objective research productivity and SNI Level found in all groups here studied might be attributed perhaps to some professors being quite effective in graduating students as PhDs, another of the elements included in the specific evaluation criteria immediately after the research products (*IEC* item III, 3.4b). The discordance between scientific productivity and SNI Level could then be explained in some cases by an exceptional contribution to the formation of highly qualified human resources or research groups, which is also a main goal of the national policy in science and technology. Yet, although it is not easy to trace the whole intellectual progeny of individual researchers, our data indicate that few PhD graduates actually become themselves researchers recognized by the SNI. And, moreover, it is difficult to understand how relatively obscure scientists might train visibly productive disciples.

### Relevance for SNI in General

As shown in Tables 4 to 9, our study reports several types of data obtained from various sources for each of 72 researchers affiliated to only one of seven general areas of knowledge in which SNI is currently divided. Such laborious approach was necessary in order to inspect the individual productivity-to-SNI Level correspondence of all professors here included, but is evidently impractical for application at a larger scale. Accordingly, our present results are valid only for Area IV and it remains to be investigated whether analogous profiles exist in other areas of SNI, or if this area stands apart as regards inconsistency in the productivity-to-SNI Level ratio of its affiliates.

Still, even the above admittedly limited outcome is a cause of concern. For should Area IV be a special instance in which scholars with widely contrasting curricula share equal official recognition and rewards, this condition would be unfair not only among colleagues who work in those same or allegedly close academic disciplines, but also in relation to those others doing research in the SNI’s remaining six general areas of knowledge. Now if, on the contrary, Area IV suffers from distortions that are more or less prevalent throughout all of SNI divisions, then the whole apparatus may be at fault and largely misleading as the presumed pattern to guide a sound career development in research. In either case the data presented here suggest a deeply anomalous situation, as already concluded from previous studies with narrower samples or scopes [46, 47, 50].

Independent indication of widespread irregularities in evaluation of scholars across all SNI general areas of knowledge comes from a rigorous non-academic source. In an inspection of SNI operation carried out in 2010 [67], the government SFA detected that the Review Committees had not specified in their written rulings which of the requisites contained in the *IEC* were determinant for the appointments granted to 396 applicants distributed in all of SNI areas of knowledge. In an attempt to find out about this, the SFA discovered that the percentage of application of the respective *IEC* varied from a maximum of 75% in Area VI (Biotechnology, Agriculture and Livestock Sciences) to just 14% in Area VII (Engineering), with Areas I (Physics, Mathematics, and Earth Sciences) and the IV studied here falling in the middle at 47%.

In addition, such official scrutiny revealed that most of the rulings issued by the Review Committees referred to cultivating human resources (theses supervised to students, teaching, creation of research groups), and none of them to introducing new knowledge into products, technology or infrastructure. Accordingly, in the conclusions of the document, the SFA advised Conacyt to review the SNI rules of operation and monitor their due enforcement, so as to make them congruent with the objectives established for the creation and statutes of the organism.

Formally ascertaining the extent of internal inconsistency in individual evaluation by the SNI would require detailed studies like the present one, with statistical random samples taken from the six remaining general areas of knowledge. Yet a much easier and faster sensor of the overall situation is immediately available. It would take just to publicly disclose all the CVUs already contained in SNI files, so that everyone interested could readily compare individual SNI Level appointments to actual productivity, as it is done in this survey. Nevertheless, this step toward full transparency has been resisted by SNI, which only publishes a 2-year outdated alphabetical list of all members showing their current SNI Level appointments, but without access to the corresponding CVUs. The official information used for the present survey could be accessed only for Area IV, upon a special permission reluctantly granted after repeated requests to the respective authorities.

### SNI as Research Policy Tool

Although not widely known among the general population, SNI constitutes a respected board of trustees commonly taken as a key reference in scholarly circles and related instances of government [69] (pp. 57–61). Such prestige is understandable, for its clusters of arbiters include some of the most highly regarded professors, who according to supposedly strict academic procedures assess and rank the upper layers of their colleagues in an apparently pristine regular operation. Yet the present findings, upon those cited above and a host of criticism from diverse quarters throughout Mexico’s academic establishment, may cast a shade of doubt over that honorable image.

Beyond a few specific analyses and the resentful comments of disappointed scholars, however, is the issue of the cost-benefit balance of cumbersome methods for evaluating scholarly production, which is a matter of current concern even in robust economies with thoroughly mature scientific communities [70]. Conacyt was recently urged once again by the SFA [71], in direct reference to SNI and another large program created to stimulate scientific networking, to develop quantitative indicators designed to measure the direct impact of public funding for science and technology. Such appeals are justified because, although SNI shows a sustained rise in the number of members over the years as proof of success in its mission of promoting and strengthening the quality of research being carried out in the country, unfortunately this surge in the quantity of researchers has not been reflected in increased numbers of patents [67, 72], nor in indicators of a commensurate effective input of domestically generated new knowledge or technologies for addressing national needs [73]. Neither that optimism has been accompanied by the creation of a proportionate number of leading-edge research centers to accommodate the newly formed highly qualified researchers, or by compelling measures for inducing the industrial sector to incorporate these experts into their workforces. In other words, little in the current surrounding scenario seems to be geared toward the SNI very reasons for existence and purpose when it was created over 30 years ago.

### Tuning up Mexico’ Research Assessment to World Standards

Available evidence, this survey included, suggests that Mexico’s main apparatus for evaluating and rewarding the performance of its scholars is largely dysfunctional. If this appreciation is mistaken, SNI should correct it by offering to view more than rising recruitment of members and mounting numbers of publications for, as shown here, neither the alleged admission and ranking of professors according to academic merits, nor the products expected from them, seem to be reliable indicators. If, on the other hand, the problem is acknowledged, then the greater difficulty arises of what should be done quickly about it.

After 30 years of operation SNI has become so deeply ingrained in the country’s academic establishment that it might be catastrophic for Mexican science to just suppress it. Neither it would be viable to replace it with local evaluation, i.e., ranking and rewarding researchers out of the same funds but by the universities and research institutes themselves, as imagined in one of the possibilities considered for 2017 among several prospective settings for the entire Mexican system of science and technology [74]. Competing with each other for tightening budgets and already facing financial limitations, there is hardly any hope that those isolated organizations would manage to avoid the same or worse faulty practices as those now existing in SNI. Rather, as also envisioned among the future options in the work just mentioned (p. 122), the SNI should resolve to undergo a serious thorough restructuring. The question then is how.

If judgment by peers is to be preserved, as the current mechanism pretends, it should be ensured that true experts in the applicants' specific fields of work assess their academic merit. One way to achieve this, which seldom has been contemplated, takes as a model the process followed by scholarly journals and funding agencies to accept manuscripts or approve scientific projects. In this conceivable approach the SNI Review Committees, instead of arbitrating directly by themselves, would act like editorial boards to locate the best possible external and independent specialists in each particular line of research, and then basically abide by their opinions and recommendations in every case. Hundreds of manuscript submissions are managed in this way every month by top multidisciplinary journals–*Nature*, *Science*, *PNAS*, *PLOS ONE*–, and there is no reason why SNI could not carry out a similar handling with the applications filed every year. This kind of outside consultation is indeed applied already by SNI, but just occasionally and mainly among nearby colleagues of the Review Committee members, rather than after systematically looking for world-class players in the respective fields of research.

Last and more difficult, but attainable and perhaps desirable if done right, is including in the assessment protocol a semi-automatic quantitative process like those presently debated as complementary or substitutes for the traditional peer-review method. As mentioned in the introduction, the extended influence of “impact factor” and other usage metrics for research evaluation has provoked legitimate concern backed by strong reasons in many scientific circles [14–21]. Nevertheless, even in front of such wide opposition, the advantages of multi-factor computer-assisted algorithmic techniques are finding enthusiastic defenders. Therefore, mixed approaches are now being explored that combine common and alternative metrics (“altmetrics”) as a promising middle-course road for an efficient and relatively objective evaluation of research quality and output by individuals, groups, and whole institutions [3, 75–79], although the usefulness of altmetrics still remains somewhat controversial [11–13].

SNI might test and then gradually adjust a scheme of this sort, for which at least one proposal specifically tailored for the purpose has been recently advanced [80]; see also [56] for an alternative all-comprehensive design. Obviously, a thorough discussion of the matter with the Mexican scholarly community is mandatory before any decisions are made.

## Conclusions

Searching for new knowledge about both the natural universe and the human world, which for centuries was powered mainly by spontaneous curiosity and serendipity, now has turned into a crucial enterprise for the shaping of national and global futures. As with any other key endeavor, continuously assessing the advancement of this process in its ever increasing complexity is an essential factor for its sound and helpful development. How to accomplish this properly is presently a matter of intense debate.

So far peer-review continues to be the best method to determine the worth of scientific projects and products. Yet merely expanding it into a massive throughput of individual cases in front of small panels of distinguished professors, as Mexico's SNI has done to huge cost for over 30 years, is apt to get seriously distorted as seen in this survey and therefore hardly the best road to follow for large numbers of researchers, let alone at a national scale. For in such situations “peers” seldom are real experts in the specific fields of work being examined, while time pressure turns “reviewing” into skimming so that evaluating becomes counting, i.e., looking for numbers rather than actual intrinsic values.

The overall impact of a scientist's oeuvre is perhaps best measureable today by an automatized combination of the various metrics now available, whereas judging the more significant qualitative elements still remains the competence of true peers. Fortunately, efficient hybrid systems including both approaches are now feasible.
